# A Perspective on PEF Synthesis, Properties, and End-Life

**DOI:** 10.3389/fchem.2020.00585

**Published:** 2020-07-31

**Authors:** Katja Loos, Ruoyu Zhang, Inês Pereira, Beatriz Agostinho, Han Hu, Dina Maniar, Nicolas Sbirrazzuoli, Armando J. D. Silvestre, Nathanael Guigo, Andreia F. Sousa

**Affiliations:** ^1^Macromolecular Chemistry & New Polymeric Materials, Zernike Institute for Advanced Materials, University of Groningen, Groningen, Netherlands; ^2^Key Laboratory of Bio-based Polymeric Materials Technology and Application of Zhejiang Province, Ningbo Institute of Materials Technology and Engineering, Chinese Academy of Sciences, Ningbo, China; ^3^Departamento de Química, CICECO – Aveiro Institute of Materials, Universidade de Aveiro, Aveiro, Portugal; ^4^Institute of Chemistry UMR 7272, Université Côte d'Azur, Nice, France

**Keywords:** PEF, poly(ethylene 2,5-furandicarboxylate), 2,5-furandicarboxylic acid, green synthesis, biodegradation, nanomaterials, recycling, packaging applications

## Abstract

This critical review considers the extensive research and development dedicated, in the last years, to a single polymer, the poly(ethylene 2,5-furandicarboxylate), usually simply referred to as PEF. PEF importance stems from the fact that it is based on renewable resources, typically prepared from C6 sugars present in biomass feedstocks, for its resemblance to the high-performance poly(ethylene terephthalate) (PET) and in terms of barrier properties even outperforming PET. For the first time synthesis, properties, and end-life targeting—a more sustainable PEF—are critically reviewed. The emphasis is placed on how synthetic roots to PEF evolved toward the development of greener processes based on ring open polymerization, enzymatic synthesis, or the use of ionic liquids; together with a broader perspective on PEF end-life, highlighting recycling and (bio)degradation solutions.

## Introduction

In decades, we have witnessed the quest for renewable alternatives to fossil-based monomers and polymers, mainly fueled by the announced dwelling of fossil resources (Williams and Hillmyer, 2008; Gandini, 2011; Mathers, 2012). Science has turned to biomass, exploring for example carbohydrate feedstocks, lignin or vegetable oils to design new building-blocks to polymer synthesis (Türünç and Meier, 2013). Such a trend has been exhaustively exploited within carbohydrates' biorefinery using C6 sugars (e.g., D-fructose feedstocks) targeting the “DOE top” (Bozell and Petersen, 2010) 5-hydroxymethylfurfural (HMF) or 2,5-furandicarboxylic acid (FDCA), typically obtained via selective oxidation of HMF intermediates (Tong et al., 2010) and references therein). Their production at an industrial scale has been implemented, paving the way for the use of furanic compounds in polymer science.

Among the myriad of polymers developed so far, within the rationale of renewable based resources, poly(ethylene 2,5-furandicarboxylate) (PEF)—synthesized from FDCA and ethylene glycol—is definitely an exceptional polymer with high-performance properties. PEF has obtained wide recognition mainly due to its resemblance to commercial poly(ethylene terephthalate) (PET) (Gandini et al., 2009; Sousa et al., 2015; Papageorgiou et al., 2016; Guigo et al., 2019) and its suitability for many general applications, especially in the packaging of carbonated drinks (Avantium, 2020) due to its enhanced carbon dioxide gas barrier properties (Burgess et al., 2014b; Araujo et al., 2018). PEF is 31 times less permeable to CO_2_ than PET (Burgess et al., 2016).

Other high-performance properties, including the thermal and mechanical behavior of the amorphous and/or semi-crystalline form, have been extensively reported (Gandini et al., 2009; Jiang et al., 2012; Knoop et al., 2013; Tsanaktsis et al., 2015b; Terzopoulou et al., 2017; Van Berkel et al., 2018). Semi-crystalline PEF relevant properties include melting (*T*_*m*_), glass (*T*_*g*_) and thermal stability temperatures of ~ 210–215°C, ~ 75–80°C and 350°C, respectively (Gandini et al., 2009). PEF exhibits a higher Young's modulus than PET (*ca*. 2.0 and 1.3 GPa, respectively) (Knoop et al., 2013; Van Berkel et al., 2018), which results in a mechanically more resilient material for final applications. Concomitantly properties related to PEF quiescent and strain-induced crystallization, glass transition, and molecular mobility have also been investigated providing deep insights into processing technologies.

Besides renewability, other aspects of green polymer chemistry have also been addressed, mainly the green synthetic roots (Loos, 2010) by ring opening polymerization (Carlos Morales-Huerta et al., 2016; Rosenboom et al., 2018), or enzymatic-assisted synthesis (Jiang et al., 2014, 2015a,b, 2016; Maniar et al., 2018; Skoczinski et al., 2020), despite the fact that the main synthetic route typically used for PEF production is a two-stage melt polymerization approach carried out under hard conditions and using metal-catalysts (Sipos, 2010; De Jong et al., 2012; Jiang et al., 2012; Jong et al., 2012; Ma et al., 2012; Knoop et al., 2013; Codou et al., 2014; Matsuo et al., 2014; Papageorgiou et al., 2014; Thiyagarajan et al., 2014).

**Figure 1:**
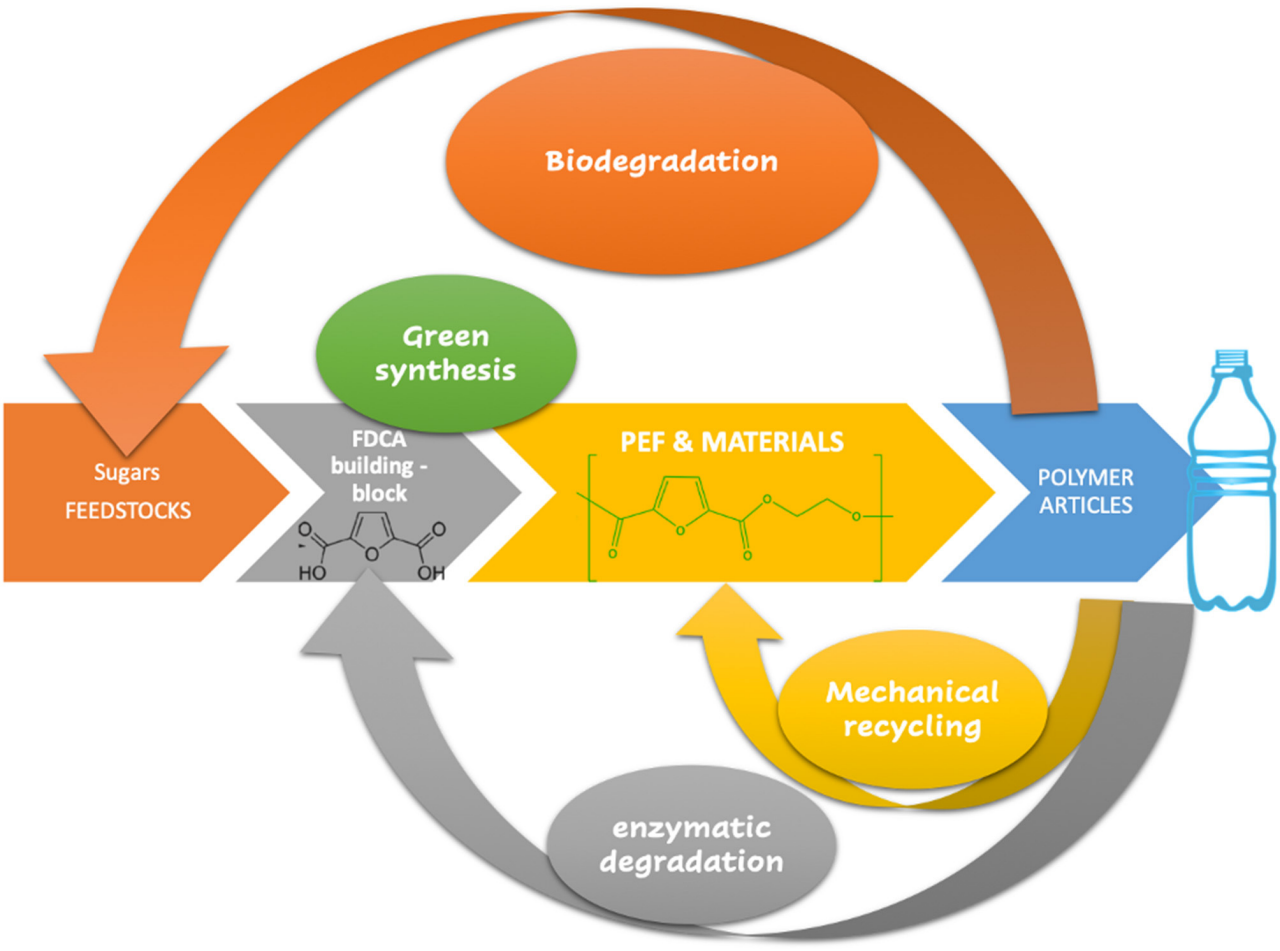
PEF sustainable approach to the circular polymers.

Last but not least, at the beginning of the twenty-first century the world awakened to the global emergency created by waste plastic debris pollution and therefore, the polymers' end-life management (recycling, biodegradation) is quickly gaining more and more importance, and the concept of the *circular polymer* and the need to address the UNs Sustainable Development Goals (UN, 2020) have never been more pertinent than today. In this regard, PEF developers have investigated both recycling (EBPB, 2017) and composting under accelerated conditions (Gruter, 2019) showing promising results, although for the latter, environmentally realistic conditions are still needed. PEF degradation carried out by pyrolysis or using enzymatic conditions are promising solutions that are currently under development (Matos et al., 2014; Pellis et al., 2016; Weinberger et al., 2017a,b; Austin et al., 2018). Figure 1 highlights these trends.

The trends highlighted in the article provide a critical overview of the development of the *work in progress*, targeting a more sustainable PEF: (i) green synthetic roots are considered; (ii) the efforts made to deeply understanding PEF's physical and chemical properties and related processing constraints and applications; (iii) nanomaterials preparation and characterization; and finally (iv) a broader perspective of PEF end-life, focused on recycling and (bio)degradation to mitigate the negative influence of (potential) future waste residues to promote a more sustainable development of PEF and to render PEF circular.

## PEF Synthesis: The Quest for Greener Roots

The first reported synthesis of PEF is almost contemporary to PET, dating back to 1946, and was patented by the Celanese Corporation of America (Drewitt and Lincocoln, 1946), following a bulk polytransesterification reaction at high temperatures (above 200°C) and applying a high vacuum. Contributions on PEF synthesis are thereafter scant, only re-starting again in 2009 with the work of Gandini et al. (2009) using a polytransesterification reaction of *bis*-(hydroxyethyl)-2,5-furandicarboxylate catalyzed by antimony(III) oxide.

The synthetic routes investigated so far for PEF production include several polycondensation and polytransesterification approaches. A two-stage melt polyesterification approach has been adapted in many studies, including a patented one (Sipos, 2010; De Jong et al., 2012; Jiang et al., 2012; Jong et al., 2012; Ma et al., 2012; Knoop et al., 2013; Codou et al., 2014; Matsuo et al., 2014; Papageorgiou et al., 2014; Thiyagarajan et al., 2014). An in-depth systematic study on the parameters affecting this two-stage polymerization, i.e., starting monomer, temperature, and especially the catalyst used, has been published by Gruter et al. (2012). They discovered that a yellow discoloration of the PEF occurred when the polymerization temperature was increased up to 260-280°C, and dibutyltin(IV) oxide was used as the catalyst. Gubbels et al. (2013) reported similar discoloration problems for some FDCA-based polymers and suggested that it was due to sugar impurities in FDCA, side reactions (e.g., decarboxylation), or due to additives such as the catalyst. Whilst metal catalysts (Sb, Ti, Ge, and Sn) perform well in PEF synthesis, the complete removal of residual metals is difficult and thus also has a negative impact on the polyester properties (coloration, thermal instability, decreased electrical performance, and potential environmental and health problems) (Finelli et al., 2004; Jiang et al., 2012; Burgess et al., 2014b; Huang et al., 2014; Papageorgiou et al., 2016; Wu et al., 2016). Due to these issues, the synthesis of PEF via a conventional polyesterification route remains challenging.

**Scheme 1:**
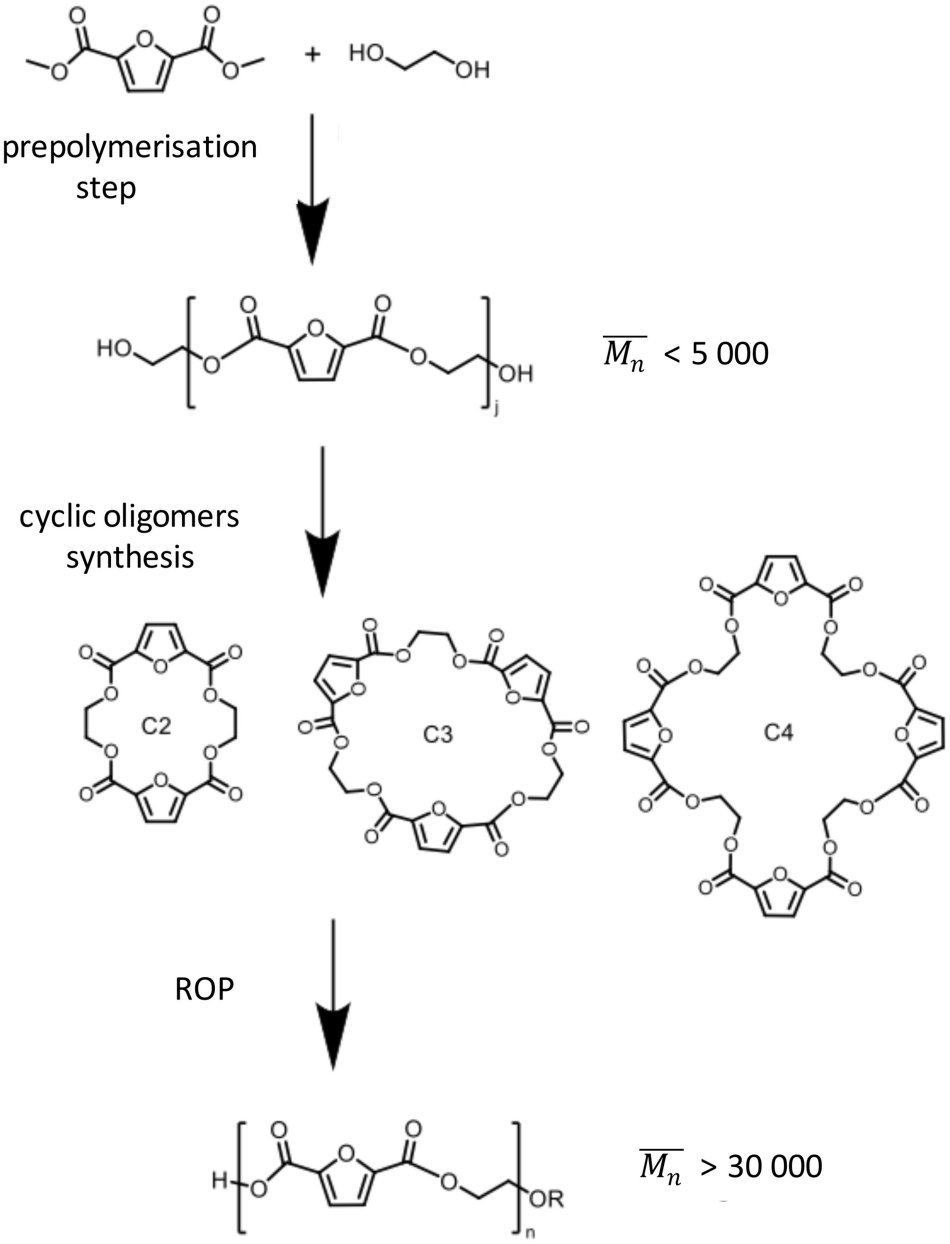
PEF synthesis via the rapid ring-opening polymerisation (ROP) approach developed by Rosenboom et al. (2018).

In an effort to render PEF synthesis more sustainable, as well as to address the above-mentioned issues, various studies have been reported (Table 1). For example (Rosenboom et al., 2018) have successfully resolved the degradation and discoloration problem by using a rapid ring-opening polymerization (ROP) technique (Scheme 1), which takes place using FDCA cyclic oligomers. They prepared bottle-grade PEF (>95% conversion with number-average molecular weight ($\bar{M_{n}}$) higher than 30 kg mol^−1^, uncolored) within <30 min, through which the usual observed degradation and discoloration was avoided. Like PET, ROP-derived PEF possess similar superiority to polycondensation-derived PEF: a higher glass transition temperature (73 vs. 85°C), which provides better room temperature thermal stability; a lower melting point (260 vs. 220°C), which can reduce the energy demand in processing steps; a higher tensile strength (± 50% higher, 50 vs. 76 MPa) and a higher Young's modulus (± 70% higher, 1.1 vs. 1.9 GPa), which results in a mechanically more resilient material for final applications. Compared to conventional polycondensation, in which high energy consumption is spent due to the high vacuum power used over long reaction times, Rosenboom et al. have established a greener and economically competitive ROP route. In addition, Carlos Morales-Huerta et al. (2016) reported a successful synthesis of the corresponding cyclic oligomers as a PEF precursor by using the non-metal catalyst 1,4-diazabicyclo[2.2.2]octane (DABCO).

**Table 1:** Summary of the main greener synthetic conditions reported for PEFs and related properties.

**Polymer**	**Experimental conditions**	$\bar{M_{w}}$	$\bar{M_{n}}$	**Ð**	**Yield (%)**	**Other properties**	**References**
**ROP**							
PEF	Bu_2_SnO; SnOct_2_; 300°C; 30 min	20,300- 79,200	9,000–40,400		≥95	Uncolored	Carlos Morales-Huerta et al., 2016; Rosenboom et al., 2018
**IONIC LIQUID—ASSISTED CATALYSIS**							
PEF	Imidazolium-based IL; 240–260°C; 3–9 h	101,100–51,100	22,700–41,900	2.25–2.65	−	−	Qu et al., 2019
**ORGANIC NON—METAL CATALYSIS**							
PEF	DBU; 170–240°C; 11–13 h					Intrinsic viscosity: 0.54 dL g^−1^	Wu et al., 2016
**ENZYMATIC—ASSISTED CATALYSIS**							
Poly(2,5-furandicarboxylate)s:	CALB; 80–140°C, 74 h, vacuum (2 mmHg) or diphenyl ether	800–48,700	500–10,100	1.10–2.05	30–97		Jiang et al., 2015b
Poly(2,5-furandimethylene)s	CALB; diphenyl ether; 80°C; 72 h; vacuum (2 mmHg)	2,700–3,900	2,100-2,400	1.29-1.77	60–74		Jiang et al., 2014
Poly(2,5-furandicarboxylate)-co-poly(2,5-furandimethylene)s	CALB; 80–95°C; 74 h; diphenyl ether, vacuum (2 mmHg)	1,500–35,400	1,400–16,100	1.07–3.21	14–96		Maniar et al., 2019
Furanic-hetero atom aliphatic polyamides	CALB; 90°C; 72 h; bulk, vacuum (30 mmHg) or toluene	14,900–16,600	6,400–8,000	2.07–2.35	26–93		Maniar et al., 2018
Poly(aliphatic furanamide)s	CALB; 90°C; 72 h; N_2_, vacuum (100–450 mmHg); or toluene	7,600–54,000	4,100–7,600	1.62–4.86	7–70		Jiang et al., 2015a
Poly(aliphatic furanamide)s	CALB; 90°C; 72 h; toluene	15,800–48,300	9,500–13,400	1.28–3.60	<50		Jiang et al., 2016

*$\bar{M_{w}}$ = weight-average molecular weight; $\bar{M_{n}}$ = number-average molecular weight; Ð = dispersity*.

Qu et al. (2019) recently undertook different approaches but also aimed at developing a more environmentally benign process. They reported the polycondensation of ethylene glycol with FDCA using eco-friendly metal-free ionic liquids (IL) as catalysts. Various imidazolium (C_n_MIM) based ILs were used in the study. They found that the solubility between ILs and monomers was the main factor that affects the catalytic activity. For PEF polymerization, they discovered that C_n_MIM-based ILs with stronger electronegativity and proton-donating ability are more efficient catalysts. PEF with the highest molecular weight ($\bar{M_{n}}$ = 52.5 kg mol^−1^) was obtained using [C_2_MIM]BF_4_ as an efficient, selective, and eco-friendly catalyst. In addition, Wu et al. (2016) investigated PEF synthesis using an organic non-metal catalyst (1,8-diazabicyclo[5.4.0]undec-7-ene, DBU) via polycondensation and a moderate molecular weight (intrinsic viscosity 0.54 dL g^−1^) was achieved.

**Scheme 2:**
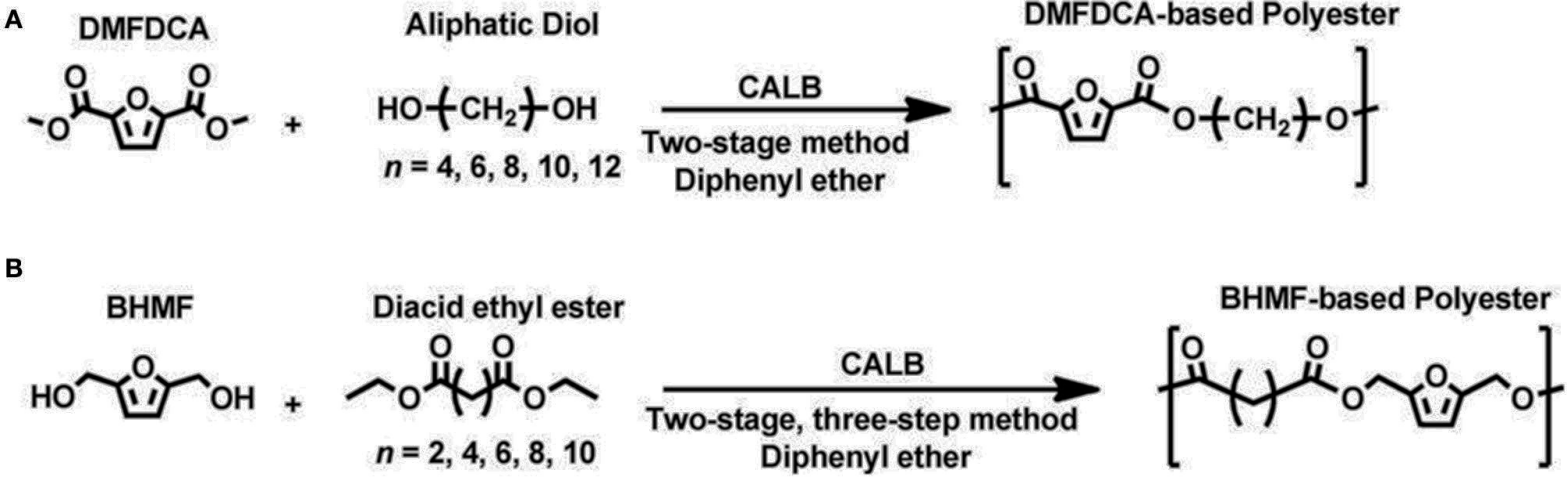
Lipase-catalyzed polycondensation of **(A)** DMFDC and aliphatic diols, and **(B)** BHMF and diacid ethyl esters developed by Maniar et al. (2019). Reproduced with permission from Maniar et al. (2019). Published by Wiley-VCH Verlag GmbH and KGaA.

With a similar aim for greener roots, Loos et al. reported the use of enzymes as a catalyst for the synthesis of various PEF-related polymers from dimethyl 2,5-furandicarboxylate (DMFDC) or 2,5-bis(hydroxymethyl)furan (BHMF) (Jiang et al., 2014, 2015b; Skoczinski et al., 2020) (Scheme 2). A high molecular weight furanic-aliphatic polyester ($\bar{M_{n}}$ = 23.7 kg mol^−1^) was successfully obtained from polycondensation of DMFDC with 1,10-decanediol (1,10-DDO), catalyzed by an immobilized *Candida antartica* Lipase B (Novozyme 435 or CALB) (Jiang et al., 2015b). Among the different diols tested, they found that the enzymatic polymerization delivered higher molecular weight products when longer-chain α,ω-aliphatic linear diols were used. Interestingly, in the enzymatic polymerization of BHMF with different diacid ethyl esters, only low molecular weight products ($\bar{M_{n}}$ = 2 kg mol^−1^) were obtained. They explained that this is due to the etherification side-reactions. Recent work from the same group has shown the feasibility of furan-based co-polyester production via enzymatic polymerization (Maniar et al., 2019). They investigated the impact of aromatic unit content on enzymatic co-polymerization of DMFDC, BHMF, aliphatic linear diols, and diacid ethyl esters. The molecular weight of the resulting furan co-polyester was found to be restricted by the incorporation of aromaticity in the backbone. These results imply that the catalytic activity of the enzyme still depends on the structural compatibility of the active site and the monomer transition state. Furthermore, a vast array of work on the enzyme-catalyzed synthesis of other furan-based polymers was also reported (Jiang et al., 2015a, 2016; Maniar et al., 2018) including furan based polyester diols with excellent end group fidelity for further polycondensation, (e.g., polyurethanes) (Skoczinski et al., 2020). With regard to the sustainability of the whole polymerization process and to address the issues in conventional PEF production, these reported studies have proven their importance as powerful approaches. Finally, these findings provide a fundamental background to designing better pathways (including enzyme engineering) for sustainable renewable-based PEF (as well as other furan based (co)polymers) production in the future.

## Structure-Properties Relationships

### Chain Conformation and Mobility

PEF has an interesting set of properties comparable to commercial benchmark aromatic polyesters, such as PET or poly(butylene terephthalate) (PBT) as previously highlighted in the Introduction. The main differences between these polyesters' properties are related to the unique features of the FDCA ring. First, it is worth mentioning that the furan ring has a lower aromaticity compared to the benzene ring or other heterocycles such as thiophene. The counterbalance is that the dienic character of the furan ring is more marked compared to other aromatics. Indeed, the oxygen of the furan ring is sp^2^ hybridized with one of its lone pairs involved in the resonance with the two π bonds. The remaining lone pair of the oxygen is involved in a p-orbital which confers to the furan ring in a dipolar moment of 0.70 Debye directed from the ring to the heteroatom (Marino et al., 1971). This favors dipolar interactions.

**Scheme 3:**
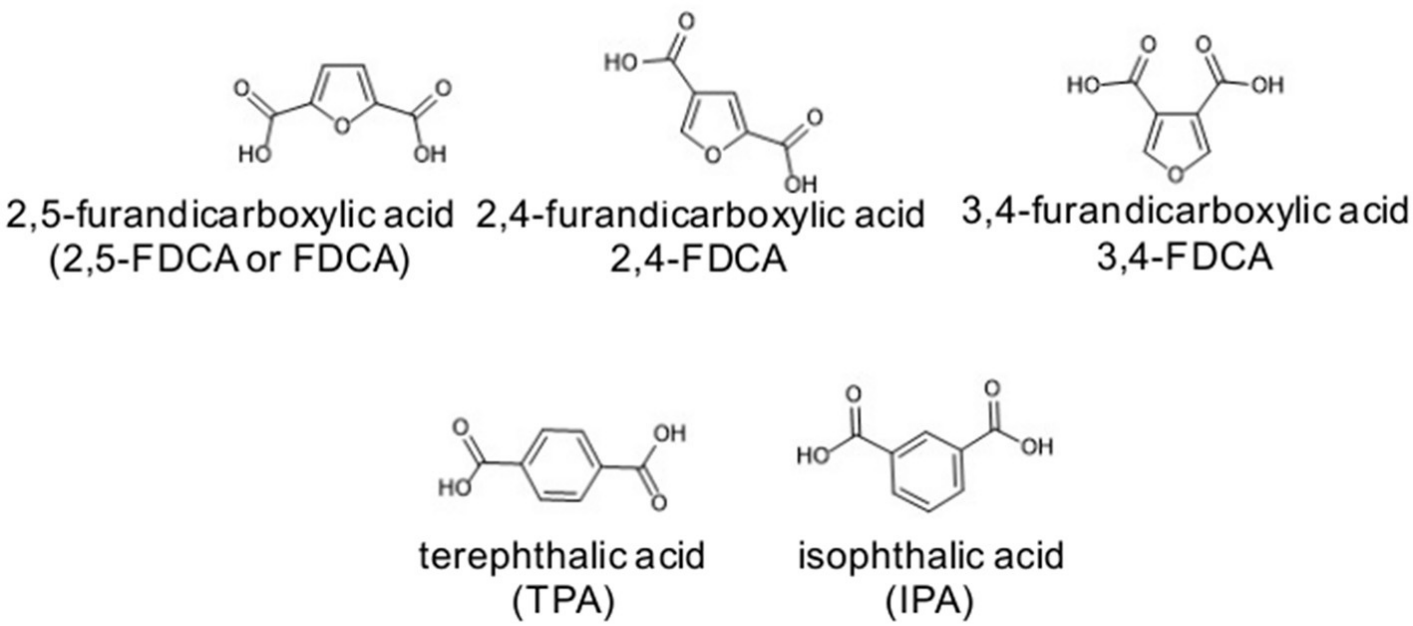
Chemical structures of 2,5-FDCA, 2,4-FDCA, 3,4-FDCA, TPA, and IPA.

Additionally, the basic structural features of the 2,5-FDCA monomer compared to its isomers, the 2,4- and the 3,4-FDCA, and also to terephthalic (TPA) and isophthalic (IPA) acids (Scheme 3) are quite singular, and thus also the polymers thereof (Thiyagarajan et al., 2013, 2014; Araujo et al., 2018; Nolasco et al., 2020). Single crystal X-ray diffraction studies have shown that the 2,5-FDCA isomer is a much less linear molecule. In fact, the projected angle between the C1-C2 bond and the C5-C6 bond of 2,5-FDCA (129°) is closer to IPA (120°) rather than to TPA (180°, i.e., linear) (Thiyagarajan et al., 2014). Indeed, this non-linear alignment, as well as the lower aromaticity described above, contribute both to the lower covalent strength of the chain axis in PEF compared e.g., to PET. Regarding size, the head-to-tail interatomic distance between the carboxylic groups in 2,5-FDCA is 4.83 Å which is 16% shorter than in TPA (i.e., 5.73 Å) (Wu et al., 2011).

**Figure 2:**
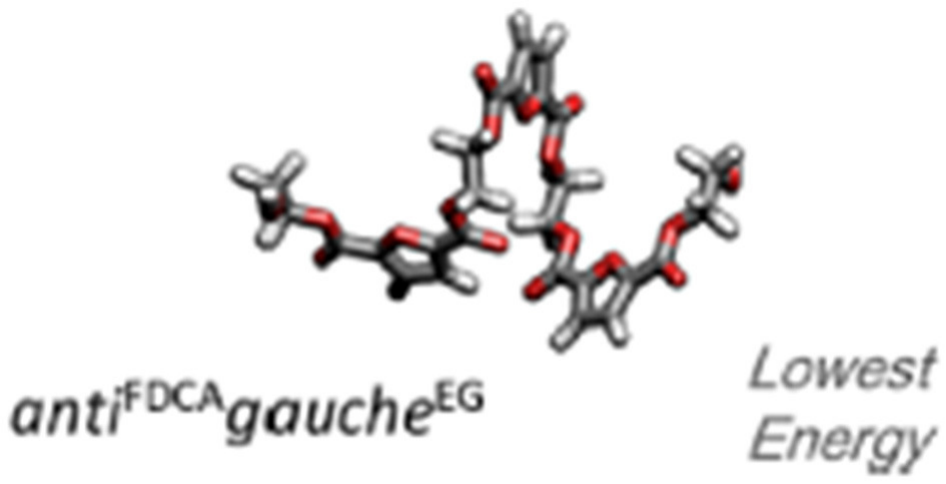
Optimized molecular geometry of a possible PEF oligomer at the B3LYP/6311+G(d,p) level of theory, in vacuo. Adapted with permission from Araujo et al. (2018). Copyright (2018) American Chemical Society.

From a conformational point of view, the 2,5-furandicarboxylate moieties in PEF can adopt two conformations: either an *anti* or a *syn* conformation where the carbonyl oxygen points away from, or in the direction of, the furanic oxygen, respectively. On the other hand, the ethylene glycol (EG) moieties in the PEF unit also have two possible conformations: *trans* and *gauche*, indicating a 180° or a 60° dihedral angle of the EG fragment, respectively. *In the amorphous regions*, according to *ab-initio* calculations of PEF oligomers, *in vacuo*, the most energetically favorable conformation is the *anti*^FDCA^ - *gauche*^EG^ coiled-helix conformation (Figure 2) (Araujo et al., 2018). In the same way, Fourier transform infrared (FTIR) data confirmed that the *anti*^FDCA^ - *gauche*^EG^ dominates the PEF amorphous structure, while the (weak) contribution of the *syn* and *trans* conformations were also visible as the sign of some statistical distributions of all possible conformations (Araujo et al., 2018).

It is worth mentioning that the coiled-helix conformation does not maximize chain packing efficiency and consequently PEF can, then, be considered a stronger glass-forming liquid (according to the Angell classification) compared to other polyesters (Bourdet et al., 2018). Moreover, the absence of linearity in PEF and the related coiled-helix conformations also explain why (Burgess et al., 2014b) and (Codou et al., 2016) have found that the free volume in the PEF glassy state is higher than in PET. This is somehow a PEF paradox since, as mentioned above, the segmental mobility is more restricted in amorphous PEF than in amorphous PET. In that respect, the size of the cooperative rearranging regions (CRR) gives a better idea of the mobility patterns since it is taking into account both the segmental mobility [linked to the glass transition temperature (*T*_g_)] and the relaxation strength. The latter is linked to the variation of the heat capacity during the glass transition, and thus, with the amount of free volume. From mechanical and thermal analyses, it appears that the CRR sizes of PEF and PET are comparable (Van Berkel et al., 2018). PEF has a higher mobility constraint which is somehow compensated by its higher free volume and, therefore, its larger relaxation strength compared to PET. In the same line, the dielectric strength of PEF is higher than the same parameter for PET (Bourdet et al., 2018).

### Crystal Structure and Crystallization Behavior

The crystalline structure as well as the crystallization process of PEF has motivated in-depth studies on the subject (Kazaryan and Medvedeva, 1968; Mao et al., 2016), mainly because the quiescent or strain induced crystals (SIC) that can be formed during processing have an impact on processing and also on the ensuing properties of the final materials. The seminal quiescent crystal structure of PEF proposed by Kazaryan and Medvedeva (1968) in 1968, was triclinic with a cell volume of 388 Å^3^. In 2016, Mao et al. (2016) reported, for uniaxially stretched PEF, a monoclinic unit cell containing two PEF chains, with a cell volume of *ca*. 774.6 Å^3^. Both authors have found a crystal density of around 1.565 g.cm^−3^ which is higher than the crystal density of PET (1.455 g cm^−3^). Very recently, Forestier et al. (2020) identified the families of the crystalline planes and they concluded that strain-induced crystals and thermo-activated crystals present similar crystalline organization.

**Figure 3:**
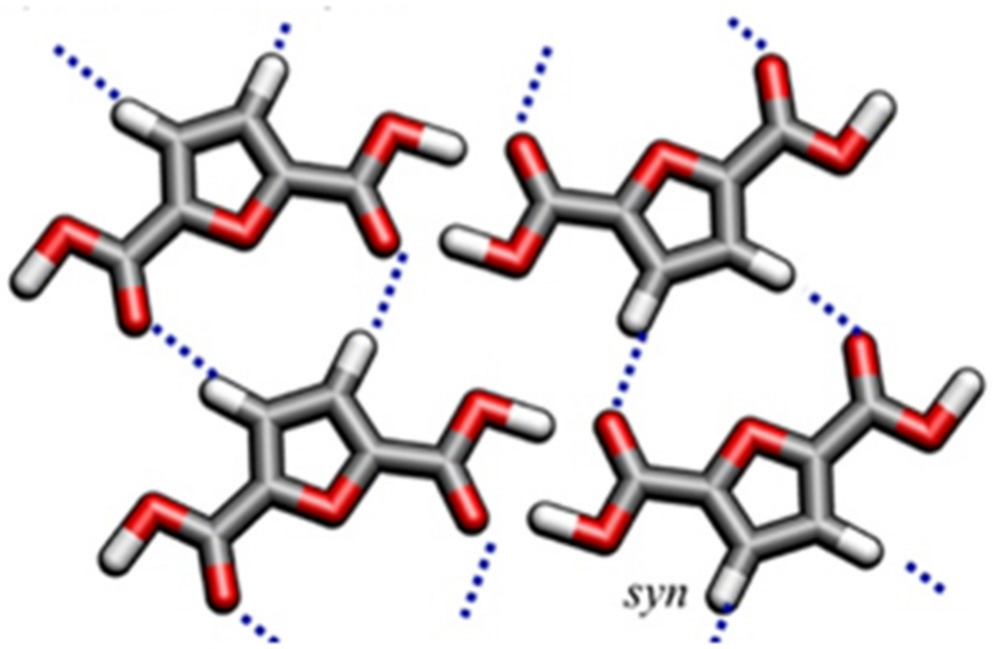
Molecular model for the *syn* conformer of 2,5-FDCA at the B3LYP/6-311++G(d,p) level. Reproduced with permission from Araujo et al. (2018). Copyright (2018) American Chemical Society.

From a conformational point of view, the occurrence of crystals (solvent-induced or from cold crystallization) in PEF induces significant changes in the conformational organization of chains, going from a typical *anti*^FDCA^- *gauche*^EG^ arrangement (coiled-helix) of the amorphous phases, to a preferred *syn*^FDCA^- *trans*^EG^ (extended *zigzag* arrangement) conformation in the crystalline regions (Araujo et al., 2018). Despite the large energy penalty associated with this transition, it is however counterbalanced by an array of hydrogen bonding, and thus packing and chain alignment for crystalline organization is favored. This also contributes to explaining the slower ability of PEF chains to crystallize compared to PET. Figure 3 shows the C-H…O hydrogen bonds for a *syn* 2,5-FDCA conformer.

The crystal microstructure depends strongly on the crystallization temperature (*T*_c_). In this respect, Stoclet et al. (2015) found that PEF chains crystallize into the so-called α form for *T*_c_ > 160°C, whereas for *T*_c_ < 160°C it crystallizes into a more defective α'-form. However, the α'-form can re-crystallize into the more stable α form when heated above 190°C (Tsanaktsis et al., 2015a). A third form, called the β-form, is obtained by solvent-induced crystallization (Tsanaktsis et al., 2015a).

Quiescent crystallization such as crystallization on cooling from the melt and crystallization on heating from the glass are very important during industrial processing of polymers such as in the injection molding of pre-forms or solid-state polymerization. The quiescent crystallization behavior of PEF has been studied in detail (Codou et al., 2014; Papageorgiou et al., 2014; Stoclet et al., 2015; Van Berkel et al., 2015). The PEF crystal growth rate is about one order of a magnitude slower than PET and passes through a maximum at 165°C (Papageorgiou et al., 2014; Van Berkel et al., 2015). This is an important advantage in injection molding, whereas slow crystallization from the melt is desired. The non-isothermal PEF crystallization behavior can be described by the classical Hoffman-Lauritzen equation and should also take into account extra phenomena such as crystal thickening for final stages of glass crystallization (Guigo et al., 2017). On the other hand, a change in the kinetic regime of the melt crystallization is highlighted for *T* < 170°C which can be attributed to the appearance of the α′-form in this temperature range (Codou et al., 2014). Finally, in the work of Martino et al. (2016a) a very tiny self-nucleation temperature range between 195 and 198°C, allowing the crystallization rate of PEF to increase significantly, has been identified.

**Figure 4:**
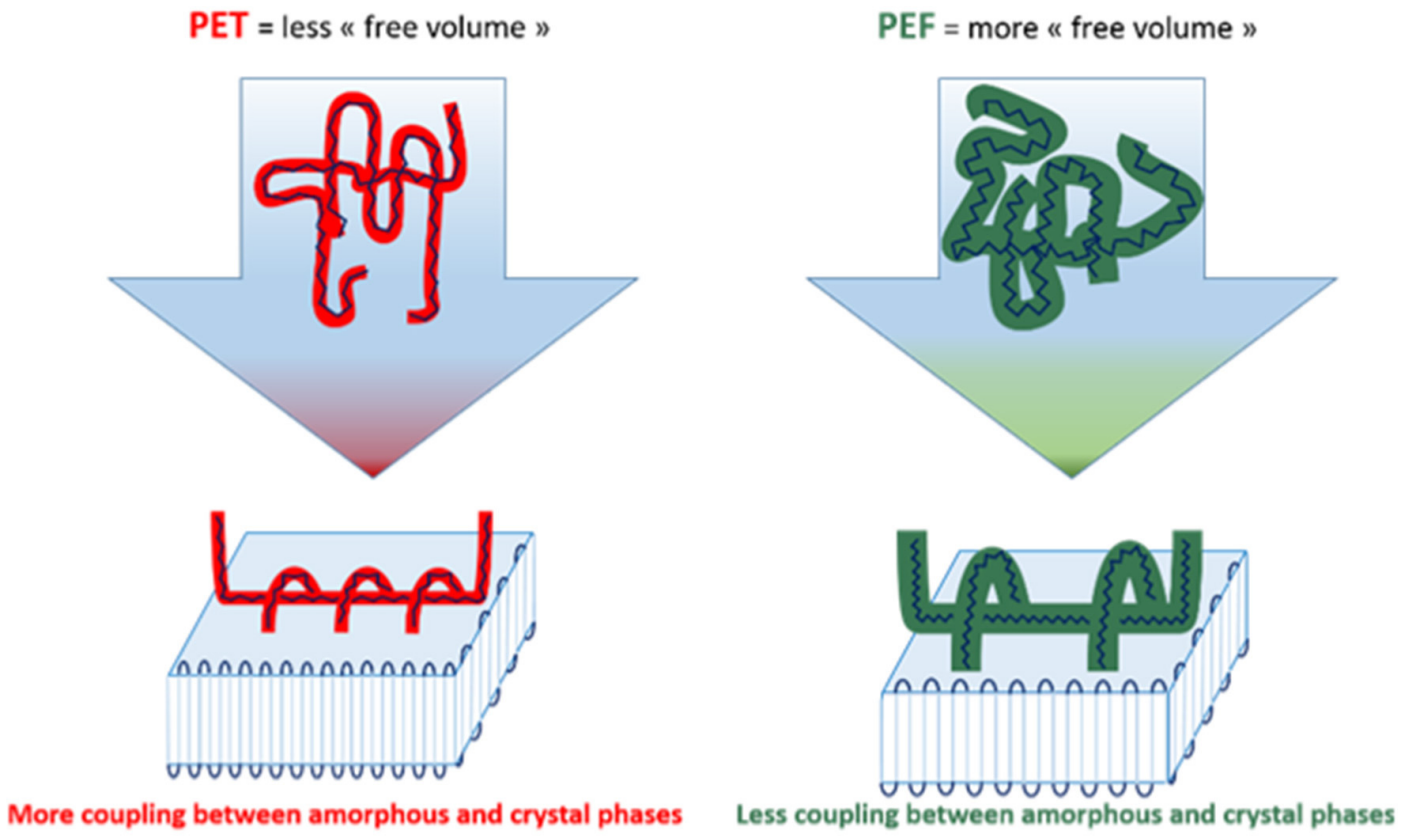
Schematic representation of the link between amorphous and crystalline phases in PET and PEF. Reproduced from Codou et al. (2016) with permission from PCCP Owner Societies.

Importantly, nucleation on cooling can be completely prevented at a relatively slow cooling rate (i.e., > 0.5 K.s^−1^). Moreover, due to the higher free volume in PEF amorphous chains, there is less coupling between amorphous fractions and the crystals compared to PET (Figure 4). Therefore, although the three-phase model is still valid for PEF, the rigid amorphous fractions (RAF) formed in the close neighborhood of crystals are less important compared to PET (Codou et al., 2016).

Conclusively it should be emphasized that PEF is prone to new microstructure developments both upon uniaxial (Stocleta et al., 2017; Mao et al., 2018; Menager et al., 2018) or biaxial stretching (van Berkel et al., 2018). SIC with a ratio up to 20% in uniaxial stretching (Menager et al., 2018) and 12% in biaxial stretching (van Berkel et al., 2018) can be formed. Compared to PET, strain hardening and appearance of SIC occurs at a higher draw ratio as a consequence of (i) the lower entanglement density in PEF *vs*. PET (Van Berkel et al., 2018) and also due to (ii) coiled helix conformations (Araujo et al., 2018). Indeed, both factors involve a higher strain to induce chain alignment and disentanglement.

### Thermal, Mechanical, and Barrier Properties

The thermal behavior of PEF was found to be relatively similar to PET. In this vein, several studies highlighted the high melting point of both PEF and PET polyesters, up to around 210–215°C, respectively, for semi-crystalline PEF samples with a number-average molecular weight ($\bar{M_{n}}$) equal to 45–55 kg mol^−1^ (Gandini et al., 2009). A glass transition of 75–80°C is typically reported; slightly higher than PET. Additionally, the thermal behavior of PEF at a high temperature was also found to be relatively similar to PET. In this vein, several studies highlighted the high thermal stability of both PEF and PET polyesters, up to around 350°C. The Bikiaris team (Tsanaktsis et al., 2015b; Terzopoulou et al., 2017) additionally studied the decomposition mechanism by Pyrolysis-GC-MS, reporting that the main mechanism involved is the β-hydrogen scission, leading to vinyl terminated and carboxyl terminated PEF chains.

From a mechanical point of view, PEF exhibits a higher Young's modulus compared to PET (*ca*. 2.0 and 1.3 GPa, respectively) (Knoop et al., 2013; Van Berkel et al., 2018). Furthermore, PEF presents a higher yield stress and higher strain rate dependence, most likely explained by additional motional constraints compared to PET. A high enough molecular weight PEF can be considered as ductile and can reach elongation at break values of around 450%. Nevertheless, its tendency toward brittleness is slightly more marked than PET. The higher yield stress in PEF is mostly linked to additional motional constraints in PEF.

#### Barrier Properties

One of the hot topics of the PEF polymer is, beyond any doubt, the barrier properties, quite relevant to packaging applications, especially when targeting food and beverage packaging. Indeed, it is interesting to note that the advantageous barrier properties of PEF over PET are most commonly underlined in the introductory sections of any new scientific publication or other communications related to PEF, and this review is no exception. This is definitely a very heavy argument in favor of PEF which, in addition to its renewable-based origin and its favorable atomic balance (i.e., C_6_ sugars give C_6_ FDCA) could make a clear-cut difference compared with e.g., *bio-*PET (Volanti et al., 2019) and other biobased polyesters such as PLA or PBS, although bioeconomic aspects will lead this fight in the end.

Burgess et al. have investigated, in detail, the sorption and transport properties in amorphous PEF (Burgess et al., 2014a,c,d, 2015, 2016). The overall gas permeability is a function of both sorption and diffusion. Interestingly, the PEF matrix has a higher CO_2_ sorption than PET due to the polar moment of the furan ring which could favorably interact with polar molecules. However, the permeability is strongly reduced i.e., x19 for CO_2_ (Burgess et al., 2016), x11 for O_2_ (Burgess et al., 2014a), and x2.1 for H_2_O (Burgess et al., 2014c,d) compared to PET. This is explained by the very limited local motions in PEF, such as the hindered furan ring flipping together with the restricted carbonyl rotations that decrease diffusion of small molecules (Burgess et al., 2014b; Araujo et al., 2018). Additionally, it is important to highlight that the lower permeability of PEF compared to PET is also preserved in biaxially-stretched samples with a barrier improvement factor of 10.4 × for O_2_ and 12.7 × for CO_2_. This provides another viable argument for the future of PEF in the bottle application field.

## PEF Based Nanomaterials

Recently, research on PEF has also pivoted into (nano)materials development (including both hybrids and nanocomposites) boosting further enhancement/control of thermomechanical properties and also in search of innovative functional properties (Lotti et al., 2016; Martino et al., 2016b, 2017; Codou et al., 2017a,b; Lam et al., 2018). To the best of our knowledge, only a few types of nanofillers have been employed so far to prepare the PEF nanomaterials, namely carbon nanotubes, montmorillonite, silver nanowires, as well as nanocellulose. Essentially all these studies have the objective to increase the thermomechanical performance of the resulting materials, to elaborate flexible optoelectronic devices, and the effective photocatalysts for anti-inflammatory/analgesic drug removal.

Lotti et al. (2016) prepared several PEF-nanohybrid materials containing 2.5 wt% of multi-walled carbon nanotubes (MWCNTs) or functionalized-MWCNTs, namely carboxyl-MWCNTs and amino-MWCNTs, or graphene oxide (GO), using a melt polycondensation approach. A slightly reduced thermal stability was noted, especially in the case of GO nanomaterials. However, they showed faster crystallization rates and increased nucleation density, which is explained by a nucleating effect of the fillers. This has resulted in smaller spherulite sizes. Most of the PEF/MWCNT nanomaterials showed a higher *T*_g_ measured by DSC and an increase of the immobilized amorphous fraction.

**Figure 5:**
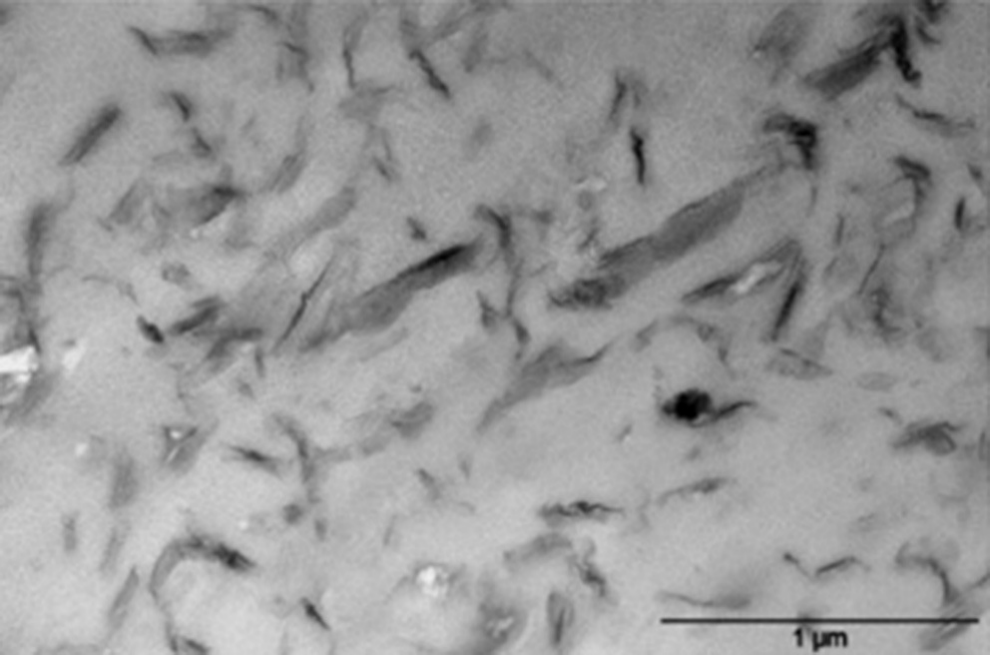
TEM image of a PEF/OMMT nanomaterial. Adapted from Martino et al. (2017) with permission from Elsevier.

Martino et al. prepared other nanomaterials based on PEF, by compounding PEF with montmorillonite modified with organophilic ammonium cations (OMMT), by a solvent casting approach employing 1,1,1,3,3,3-hexafluoro-2-propanol (HFIP) as solvent (Martino et al., 2016b), or by extrusion (Martino et al., 2017). In the latter case, dimethyl benzyl hydrogenated tallow alkyl (Closite 11) was selected to organically modify the clay (Martino et al., 2016b). Transmission Electron microscopy (TEM) (Figure 5) and the Wide-angle X-ray scattering (WAXS) patterns indicated that PEF chains tend to exhibit a predominantly intercalated-type morphology with some individual exfoliated clay platelets in the PEF/OMMT nanomaterials. A slight increase in the melt crystallization rate and a higher crystallinity was observed by DSC measurements. Indeed, the presence of the clays slightly affected the sample crystallization behavior accelerating its rate due to a nucleating effect of the OMMT clays, although no differences were observed for the *T*_*g*_ values. Thermogravimetric analysis (TGA) results showed that PEF/OMMTs have higher thermal stabilities than pure PEF, both under inert (+20°C) and oxidative atmosphere (+30°C). In fact, the increased thermal stability of the new PEF/OMMTs in thermo-oxidative conditions is compatible with the clays, acting as a barrier for the oxygen diffusion within the sample.

**Figure 6:**
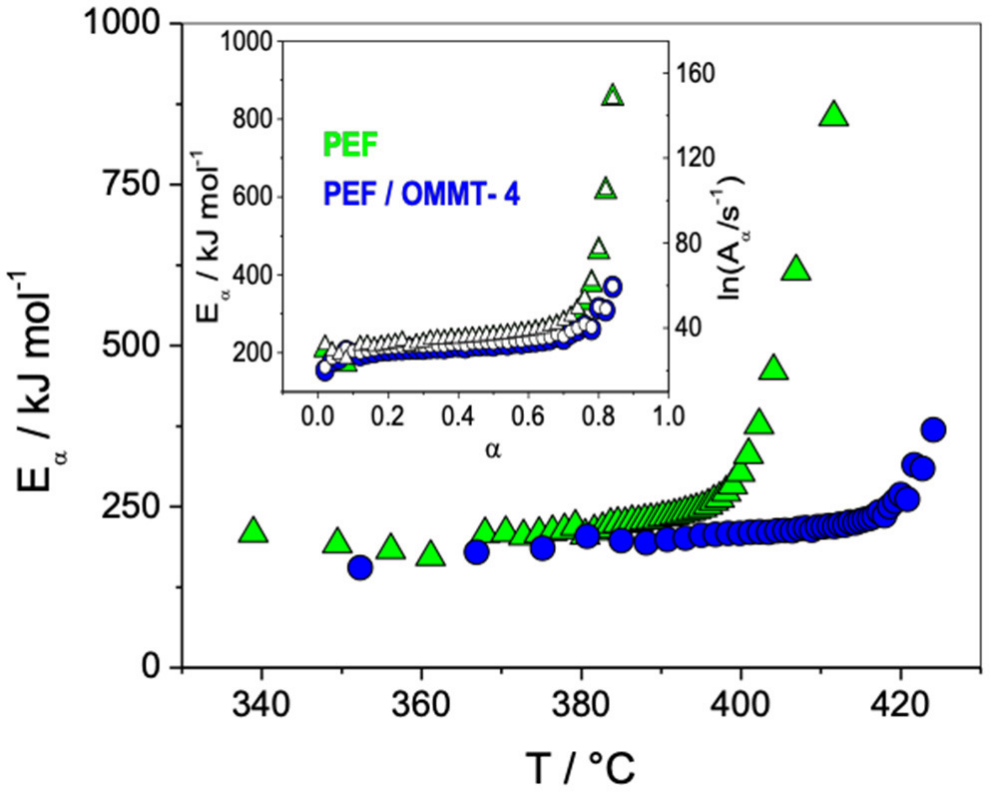
Effective activation energy (*E*_α_) dependencies vs. temperature (T) of neat PEF and PEF nanocomposite with 4% w/w OMMT (PEF/OMMT-4). Inset: Effective *E*_α_ and logarithm of pre-exponential factor [ln(A_α_)] dependencies vs. extent of degradation (α). Values are always increasing but accelerating at α ~ 0.70. At the beginning of the thermal degradation, for 0.10 < α < 0.20, the values are quasi-constant which indicates that the overall degradation mechanism is mainly governed by a single-step process, corresponding to the breakage of weaker bounds (e.g., ether bonds). Applying the (Sbirrazzuoli, 2013) method leads to the conclusion that the degradation mechanism obeys the contracting cylinder model *R*^2^. For α > 0.70, the high increase of *E*_α_ indicates that the end of degradation obeys a complex multi-step mechanism which involves simultaneous breakages of several chemical bonds and chemical rearrangement to produce low molar mass molecules. This value of 0.70 corresponds to a temperature of around 398°C for PEF and around 418°C for PEF/OMMT-4. Adapted from Martino et al. (2016b) with permission from PCCP Owner Societies.

An advanced isoconversional kinetic analysis was also performed to gain insight into the thermal degradation mechanism of these PEF/OMMTs: the effective activation energy (*E*_α_) and pre-exponential factor (ln*A*_α_) of neat PEF and ensuing nanomaterials were studied (Figure 6). This study reported similar variation in the *E*_α_ and ln*A*_α_, thus, indicating equivalent degradation mechanisms. A very important result of this study was the fact that PEF thermal degradation was slowed down in the presence of the clay. This happens because of a purely physical effect, such as a shielding effect, without modifying the intrinsic degradation mechanism of PEF. Moreover, it is well-known that polyesters can be subjected to thermal degradation or thermally induced hydrolysis during processing at a high temperature, thus this delay in degradation could represent an advantage for processing and material forming at a high temperature.

Similar conclusions can be drawn for PEF/clay hybrid materials processed via the melt extrusion method (Martino et al., 2017). Montmorillonite was also organically modified with dimethyl benzyl hydrogenated tallow alkyl or octadecylamine and organophilic sepiolite containing pending epoxy groups. However, the increase in thermal stability was lower (16–21°C) than for solvent casted materials. This is explained by the partial degradation of the sample that may occur during the extrusion cycles, as evidenced by a decrease in the molecular weight. Recently, Xie et al. (2019) also prepared PEF/OMMT nanocomposites, but via a melt polycondensation of DMF and EG in the presence of an organic montmorillonite modified with octadecyl hydroxyethyl dimethyl ammonium (DK2). Partially exfoliated structures were obtained and the PEF nanocomposites containing 2.5 wt% DK2 showed significantly improved melt crystallization, tensile modulus, and strength.

**Figure 7:**
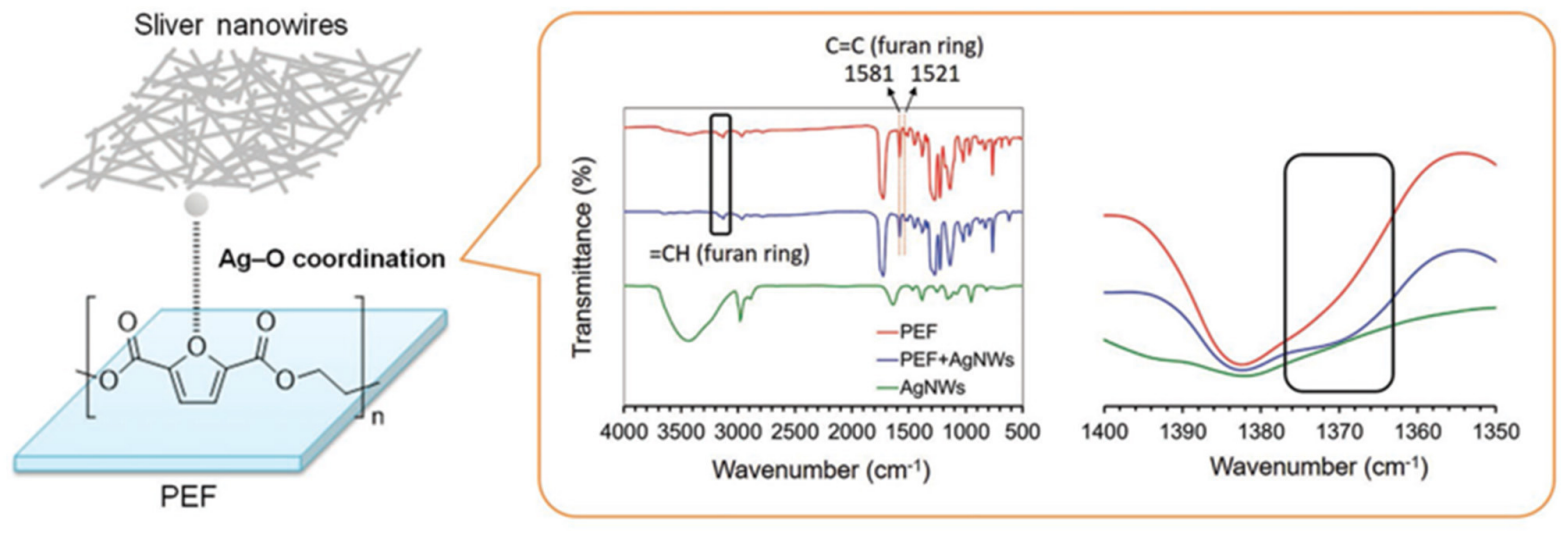
Schematic illustration of PEF/AgNWs conductive film and FTIR spectra of PEF/AgNWs and related PEF and AgNWs components. Reproduced from Lam et al. (2018) with permission from John Wiley and Sons.

In a recent work by Lam et al. (2018), flexible conductive films, with a good transparency, were successfully developed by coupling PEF with silver nanowires (AgNWs) (Figure 7). An intense interaction with AgNWs has proven to largely enhance the adhesion of AgNWs grown above, as exemplified by the superior bending and peeling durability compared with the PET substrate. Conductive PEF/AgNWs films were successfully used to obtain flexible organic thin-film transistor and organic photovoltaic (OPV). The OPV device achieved a power conversion efficiency of 6.7%, which is superior to the device based on Indium Tin oxide (ITO)/poly(ethylene naphthalate) (PEN), i.e., the ITO/PEN device. Therefore, new hybrid materials show promising applications within flexible electronic applications (Lam et al., 2018).

Other studies addressed the use of cellulose nanocrystals applied as fillers in thermoplastic nanocomposites. For packaging applications, cellulose nanocrystals have attracted significant interest due to the claim of safety and efficiency in food products. Moreover, cellulose is the reinforcement of choice in applications within bio-based materials, although still not addressed as such within PEF development. Other properties, like high specific surface area, easy surface modification, high stiffness, and low density has attracted the interest of the scientific community. In this line of thought, and most probably bearing in mind the unique features of nanocrystalline cellulose (NCC), Codou et al. (2017a,b) reported the preparation of nanocomposites based on PEF and NCC. The authors followed two approaches, one based on melt processing (Codou et al., 2017a) and, for comparison reasons, another using a solvent casting root which is considered to be an effective and simple method (Codou et al., 2017b) and which avoids thermal treatment at high temperatures which may contribute to polyester hydrolysis and molecular weight decay. However, volatile organic solvents are used with this film-preparation approach.

In the case of the novel nanomaterials prepared following the melt extrusion procedure (Codou et al., 2017a), scanning electron microscopy (SEM) images of the fracture surface of PEF/NNC showed that the micrometric cellulose flakes were dispersed in the PEF matrix and no de-cohesion was detected. Good interfacial adhesion was observed between PEF and cellulosic flakes of micrometric dimensions. The thermal degradation of PEF was not modified by the presence of NCC, contrary to what was observed with PEF/clay nanocomposites. However, cellulose favorably modified the crystallization behavior: PEF/NCC composite crystallinity is two times that of neat PEF, the presence of cellulose induced a higher crystallization rate and the nucleating effect increases with the cellulose concentration. This is reflected by the crystallization half time that decreases by about 35% when cellulose is dispersed in PEF.

**Figure 8:**
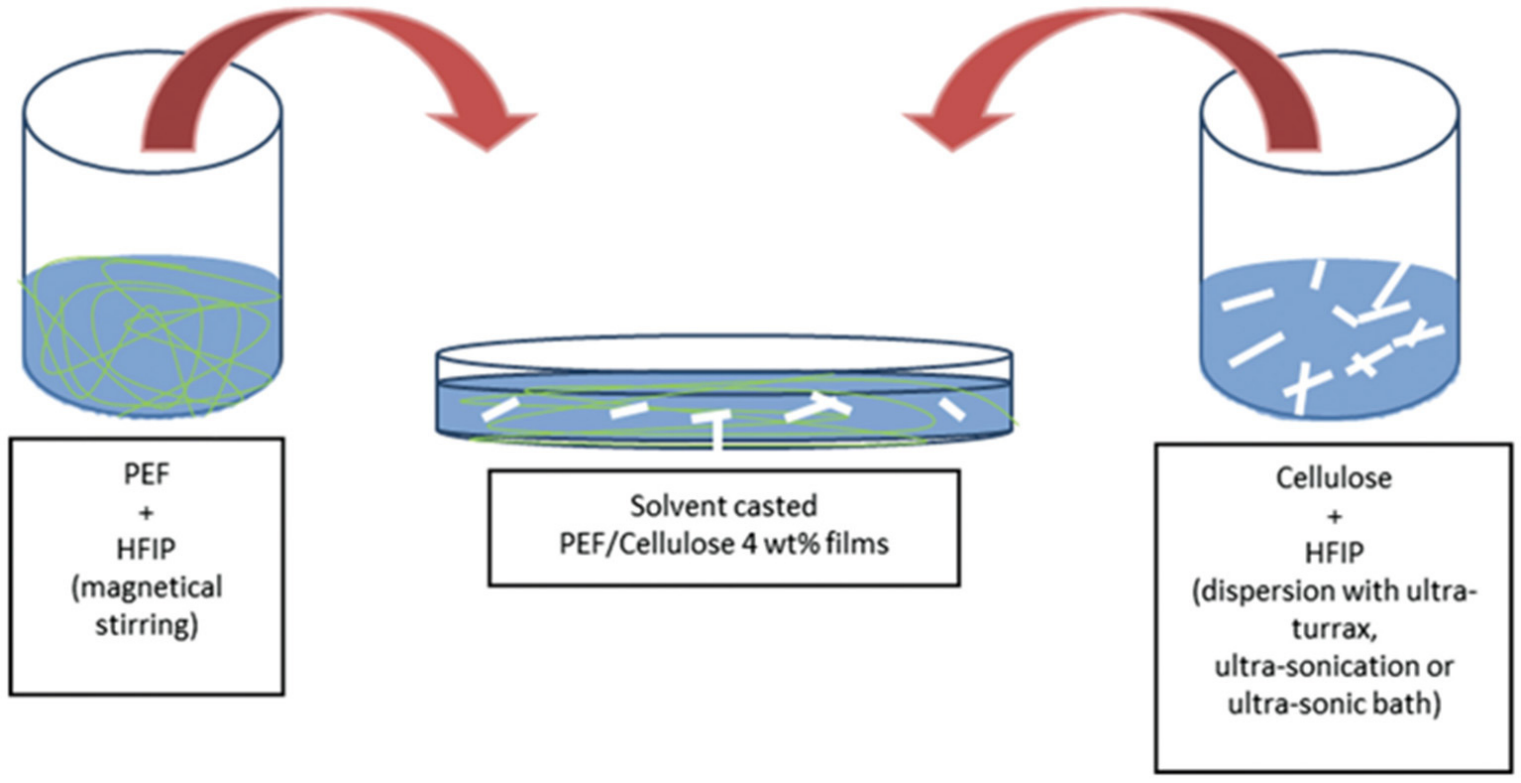
Schematic representation of the solvent casting root used to develop novel PEF/nanocellulose films. Reproduced from Codou et al. (2017b) with permission from Walter de Gruyter.

In the other case of PEF/NCC nanocomposites prepared using the solvent casting route (Figure 8) (Codou et al., 2017b), the dispersion within the polymeric matrix revealed to be a challenging objective. Different procedures to disperse NNC in HFIP, with equipment of increasing dispersive energies (ultra-sonic bath, ultra-sonication and ultra-turrax), were used. The authors observed that even after hours of treatment the NCC/HFIP suspension still appeared cloudy, revealing the presence of micrometric cellulose particles, being the ultra-sonic bath, and from a process point of view, is the most favorable to disperse cellulose in PEF. Obviously, this circumstance could be overcome by rendering cellulose more hydrophobic via chemical modification, but the authors opted to maintain a more straightforward process. Nevertheless, FTIR spectroscopy of the nanocomposites revealed some broadening of the OH- bands most probably associated to interactions between PEF and cellulose. Additionally, cellulose played a slight nucleating effect, as highlighted by DSC measurements. This result is in line with the conclusions made for the other composites prepared via extrusion (Martino et al., 2017). The thermal resistance analyzed by TGA showed a slight shift of the thermal degradation temperature to higher temperatures, *viz*. 348°C for PEF and 355-363°C for the nanocomposites.

## PEF Applications

PEF is expected to be used in many general applications, like for example in packaging of soft and alcoholic beverages (Avantium, 2020; CORBION, 2020), as already mentioned before in the barrier properties section, as well as in films applied in the packaging sector or as fibers for textiles. In this vein, several announcements of new products based on PEF have been made. One example using PEF and wood fibers publicized by the Carlsberg Group is the “Green Fibre Bottle” prototype suitable for containing beer (Carlsberg, 2019).

Until now, PEF demonstrates to be a good option for the future- *but at what cost? Does PEF prices compete with fossil-based polymers?* According to Eerhart et al. (2015), in 2015 the price of PEF had to be around 1500 $ per ton in order to compete with “giant” counterparts. Also, according to the same authors, the economic analysis performed revealed that PEF can be produced, starting from wheat, with prices ranging from 150 to 1750 $ per ton, under the conditions of large-scale production, proper price levels for by-products, and if wheat straw (used as feedstock) prices remain within 50-150 $/ton, but these figures have potential for further progress especially if more efficient sugar sources are used, if their conversion into FDCA evolves, and finally, if the PEF production process also evolves. In sum, PEF has the ability to compete with its petro-based counterparts. Importantly, PEF large-scale production through Avantium's YXY Technology was validated at pilot scale and, was announced to be ready for scaling up to a flagship plant in 2023. PEF price is a major constraint in its full penetration into the market. However, owing to PEF superior barrier properties its production and commercialization can be boosted and can benefit from specific niche markets.

## PEF End-of-Life

Nowadays, the social awareness of the negative impact of polymers', especially when they end up abandoned in land-fields or accumulate in the aquatic environment, together with more restrictive legislations are pushing industrials and scientists to look for end-life solutions. This is no exception for the PEF case. In fact, in this context, recycling as well as biodegradation are solutions highlighted by some authors to make circular-PEF.

In the quite feasible scenario of PEF commercialization and introduction in waste side streams, the reprocessing of PEF waste by physical means (gridding, shredding, melting) in order to form a new product (Grigore, 2017; Okan et al., 2019), usually simply referred as mechanical recycling, has been addressed (De Jong et al., 2012; EBPB, 2017; Kucherov et al., 2017; Terzopoulou et al., 2017; Alaerts et al., 2018). Chemically recycling, understood as the depolymerisation by hydrolysis (prompted by enzymes), alcoholized or thermally-induced (i.e., pyrolysis) into monomers or oligomers, that can be subsequently re-polymerized (Grigore, 2017; Okan et al., 2019), has also been studied (Pellis et al., 2016; Weinberger et al., 2017a,b; Austin et al., 2018). Additionally, a PEF composting test was also conducted (Gruter, 2019).

### Mechanical Recycling

There are some technical challenges that need to be overcome to efficiently recycle PEF, as previously wisely pointed out by Alaerts et al. (2018). One of these challenges is related with the fact that PEF and PET are analogous and that they can have a similar appearance and/or physicochemical properties; besides the fact that in PEF similar technical applications to PET may be found (e.g., in plastic bottles) (Alaerts et al., 2018). Therefore, their pure streams recovered in technical recycling chains, within a mechanical recycling rationale, can be economically unfeasible and/or a technically impossible task (EBPB, 2017; Alaerts et al., 2018) because the traditional washing or sink-float step techniques, do not lead to complete separation and recovering of pure streams. Most probably, considering this, the Technical Committee of the European PET Bottle Platform (EBPB), at the request of the former Avantium-BASF JV, conducted an evaluation of the effect of PEF on the PET recycling stream, and the use of near-infrared (NIR) sorting equipment to distinguish between the two polymers (EBPB, 2017) was reported. Although this could be a measure to overcome sorting, it also means that sorting centers should be equipped with NIR equipment.

On a very short report by EBPB (2017), the Association disclosed that for a PET recycling stream contamination of up to 2% of PEF (corresponding to this polymer maximum allowed market penetration) there is no negative impact on recycled PET haze, color, and other properties. De Jong et al. (2012) also briefly reported the influence on tensile behavior of up to 5% PEF on PET/PEF mixtures, extruded into standard tensile bars, and no significant effect was claimed. Moreover, predictions say that the amount of existing PEF, at least until 2021, will be as high as 0.45%, much lower than the previous percentages, hence (for now) no risk seems to appear as far as the quality of recycled PET is concerned (Alaerts et al., 2018). Nevertheless, broader and deeper development of end-life solutions are expected to be developed in the near future due to the increasing demand for polymers circularity (Kaur et al., 2018; European Commission, 2019).

**Figure 9:**
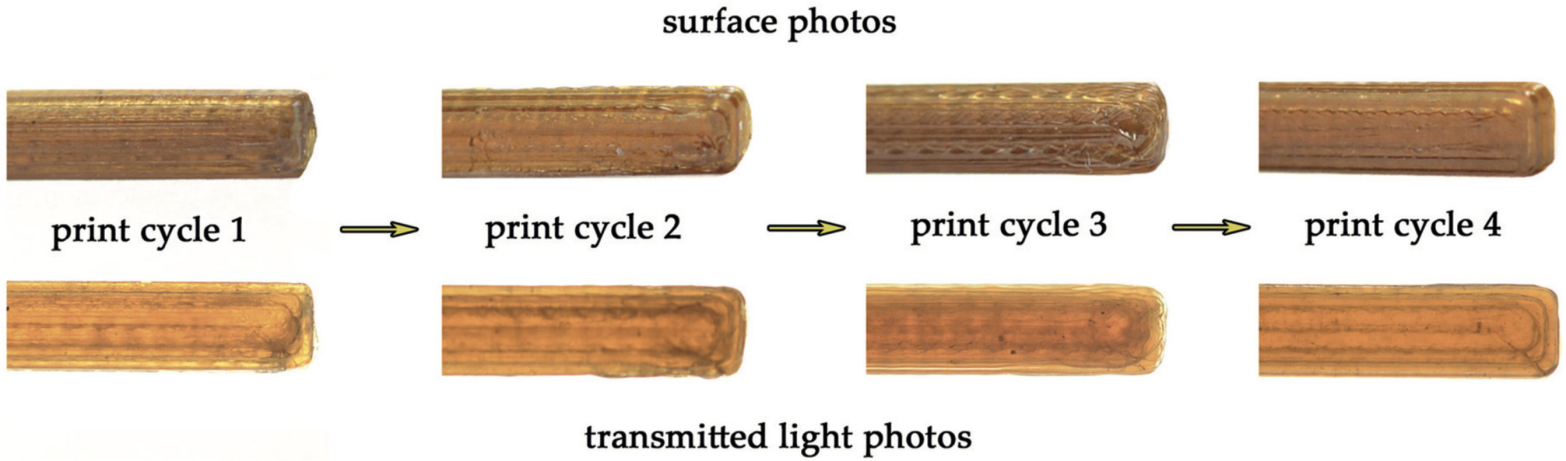
Recycling in several 3D printing cycles. Adapted from Kucherov et al. (2017) with permission from John Wiley and Sons.

Recent studies by Kucherov et al. (2017) reported the Three Dimensional (3D) printing of PEF and already studied PEF successive cycles of 3D printing and recycling (Figure 9). In this study, after four recycling cycles where PEF was extruded at 215°C, it maintained its structural integrity, unlike poly(lactic acid) (PLA), acrylonitrile butadiene styrene (ABS), and poly(ethylene glycol-co-1,4-cyclohexanedimethanol terephthalate) (PETG). Furthermore, DSC measurements revealed a glass-transition temperature and enthalpy variation of up to 6 and 40%, respectively. Moreover, an elemental analysis demonstrated no more than 0.2% changes in carbon and hydrogen PEF composition. The authors concluded, then, that due to the high thermal stability of PEF the 3D printed objects could be recycled without noticeable degradation (Kucherov et al., 2017).

More and more PEF synthetic approaches are being studied and optimized, making use of different catalysts and reaction conditions, so that it can become more sustainable and less impactful in the environment (Carlos Morales-Huerta et al., 2016; Terzopoulou et al., 2017; Kasmi et al., 2018; Rosenboom et al., 2018; Banella et al., 2019; Chebbi et al., 2019; Maniar et al., 2019). In this vein different authors tested different catalysts in PEF synthesis, and in particular (Terzopoulou et al., 2017) tested the effects of different catalysts on PEF mechanical recyclability. In this study, titanium(IV) butoxide, titanium(IV) isopropoxide, tin(II) 2-ethylhexanoate, and dibutyltin(IV) oxide were evaluated by simply carrying out three melting (250°C for 10 min) and cooling cycles of PEF, prepared using each of these catalysts. As expected, the number of carboxylic acid chain-ends increased with the number of recycling cycles as detected by FTIR spectroscopy; also, a gradual decrease in the intrinsic viscosity was observed. Titanate catalysts were responsible for a steeper increase in polymer degradation (Terzopoulou et al., 2017). Moreover, based on Pohl's method (Pohl, 1954) titanate catalysts were responsible for roughly 6-7 times higher carboxylic acid groups content (Terzopoulou et al., 2017).

### Enzymatic Degradation

PEF degradation by enzymatic hydrolysis of PEF has been systematically investigated under a range of conditions, mainly the effects of molecular weight, crystallinity, and the kind of enzyme (Pellis et al., 2016; Weinberger et al., 2017a,b; Austin et al., 2018). Pellis et al. (2016) examined the degradation of PEF powder with various molecular weights (6, 10, and 40 kDa) through cutinase 1 from *Thermobifida cellulosilytica* use. Specifically, the biocatalyst showed a high hydrolytic activity on 40 kDa PEF powder for 72 h of incubation at 50°C. The ensuing released products were identified by LC/TOF-MS, finding that the most abundant product was FDCA, followed by several related oligomers.

In 2017, the same group (Weinberger et al., 2017b) also investigated how the crystallinity and the particle size of PEF powders affect enzymatic hydrolysis. In this vein, amorphous PEF films were prepared via melt-compression at 250°C; and different percentage crystallinity PEF samples were prepared by annealing assays at 170°C for 0.5 and 1 h (10 and 20% crystallinity, respectively). Comparative studies have indicated that increased crystallinity considerably decreased the release of FDCA (especially for the 20% crystallinity). Additionally, with the same particle size, the lower molecular weight sample hydrolyzed faster. However, when the molecular mass was high enough, the influence of the particle size on the hydrolysis became negligible. Overall results indicated that the PEF films are enzymatically hydrolyzed 1.7 times faster than PET ones. In another study (Weinberger et al., 2017a), the authors adjusted the degradation parameters to achieve a 100% hydrolysis of the PEF films after only 72 h of incubation with a *Humicola insolens* (HiC) cutinase in a potassium phosphate buffer (1 M KPO, pH ~ 8) at 65°C.

Recently, researchers used newly discovered PETase, rather than cutinases, to degrade PEF (Austin et al., 2018). In this study PETase (PET-digesting enzyme) secreted by a newly discovered bacterium, *Ideonella sakaiensis* 201-F6, was engineered to degrade both PET and PEF owing to its collection of subtle variations on the surface of a lipase/cutinase-like fold.

### Miscellaneous Studies on PEF Related Polymers Degradation

Several other studies addressed the issue of promoting-degradation using polymer chemistry ability to modify PEF and, thus, to tailor its degradation rate. A predictably wider spectrum of applications for these polymers can, thus, be envisaged.

One of the main approaches studied so far was the synthesis of furanic–aliphatic co-polyesters by incorporating a third component into the PEF backbone to tailor the thermal and mechanical properties (Xie et al., 2018), as well as the degradability profile. As noted previously for PEF, the molecular weight, crystallinity, kind of enzyme, and/or hydrophilic/hydrophobic balance also played a role on copolymers counterparts (Yu et al., 2013; Terzopoulou et al., 2016; Jia et al., 2018). One such study, making use of furanic-aliphatic approach, is the work of Jia et al. (2018). These authors reported random poly(ethylene dodecanedioate-co-ethylene 2,5-furandicarboxylate) copolymers (PEDFs) with varying feed molar ratios of diacids. These polymers changed from semi-crystalline thermoplastic to the completely amorphous elastomer with a controllable hard segment (FDCA) and soft segments (dodecanedioic acid) (Jia et al., 2018). Enzymatic degradation tests carried out using lipase from *Porcine pancreas* consistently showed a weight loss increase with the increase of aliphatic units, except in the case of the copolymer with 60% of dodecanedioate moieties. This latter fact was related by the authors to the presence of “incomplete crystalline regions” (Jia et al., 2018). PEF co-polyesters incorporating different diols to endow tailored thermal and mechanical properties (and most probably also influencing degradation, although to the best of our knowledge this was not evaluated) were also synthesized. This includes those based on 1,4-butanediol (Ma et al., 2012), 2,2,4,4-tetramethyl-1,3-cyclobutanediol (Wang J. et al., 2017), 1,4-cyclohexanedimethanol (Hong et al., 2016; Wang et al., 2016). Additionally, more hydrophilic oligomeric diols were also considered, namely poly(ethylene glycol) (PEG) (Hu et al., 2018; Xie et al., 2018) and poly(tetramethylene glycol) (Xie et al., 2018).

Other works used instead, as a third comonomer, succinic acid (Yu et al., 2013; Terzopoulou et al., 2016), adipic acid (Papadopoulos et al., 2018), or sebacic acid (Wang G. et al., 2017). The Bikiaris group showed that adipate-based co-polymers have enhanced degradability, reaching near 100% weight loss in only 20 days (Papadopoulos et al., 2018); although more moderate degradability was reported for copolymers from succinic acid (Terzopoulou et al., 2016). Clearly, these copolymers' enzymatic hydrolysis positively depended on the engineered comonomers ratio and on the related flexible polymer structure of the aliphatic moieties (Soares et al., 2017), as well as on the decrease of crystallinity (Papadopoulos et al., 2018).

**Figure 10:**
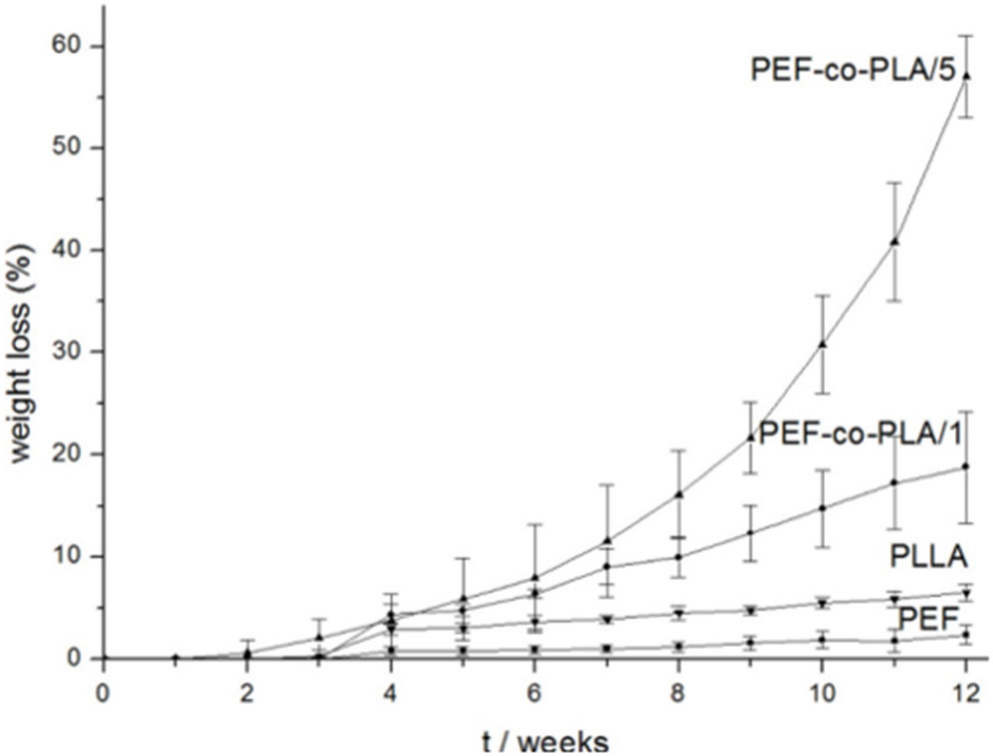
Weight loss percentage of PEF copolyesters with 27 and 93% of PLA moieties (PEF-co-PLA/1 and /5, respectively) along with hydrolytic degradation. Reproduced from Matos et al. (2014) with permission of John Wiley and Sons.

Co-polyesters based on PEF and oligomeric poly(lactic acid) (PEF-co-PLA) were prepared by Sousa's team (Matos et al., 2014) using different starting feed ratios. *In vitro* hydrolytic degradation under a phosphate buffer solution (pH 6.9), at 37°C, and water-absorption studies carried out in demineralized water, illustrated that copolyester with 29% of lactyl units possessed relevant degradability (Figure 10) and enhanced water absorption. Products following the hydrolytic degradation of these novel furanic-lactyl co-polyesters have ATR FTIR detectable free hydroxyl and carboxylic acids groups due to the occurrence of ester bond cleavage.

### Biodegradation

Biodegradation studies on PEF, together with several other renewable-origin polymers, have evolved in recent years to understand and tailor the biodegradation profile of this new polymer (Gruter, 2019). However, to go into further detail, two interconnected questions are pertinent at this point of this review: *What is biodegradation? How can biodegradation be studied?* Biodegradation is usually understood as a natural process prompted by microorganisms (Hodzic, 2004; Sinha Ray, 2013) by which organic chemicals undergo conversion to simpler compounds, (e.g., by cleavage of polymers' chains), and then mineralization and redistribution through elemental cycles such as the carbon, nitrogen, and sulfur cycles. In this regard, the enzymatic degradation studies of PEF described previously (Pellis et al., 2016; Weinberger et al., 2017a,b; Austin et al., 2018) provide only a first picture of how this polyester may behave. Typically, biodegradation is studied under composting conditions carried out at specific times, temperatures, and in a controlled microbiome environment, according to standard tests (ISO17088, 2012). In this vein, accelerated tests of PEF biodegradation by Organic Waste Systems (OWS) have been briefly reported by Avantium (Gruter, 2019). In these tests, although the results are quite promising, showing that after <9 months only most of PEF had biodegraded, in contrast to PET which persisted, the conditions used, such as a high temperature (58°C), are still not environmentally relevant. Further studies under natural conditions (such as typical aquatic and soil settings) are required to provide realistic biodegradation insights.

## Conclusions & Future Prospects

Finally, the information critically reviewed here provides a fundamental background of what is known in terms of PEF chemical and physical properties, used by industrial players to correctly plan processing technologies, avoiding for example crystallization, or, alternatively promotes a higher crystallinity thickness to post-polymerize, and above all, to provide clear evidence of PEF application within packaging, especially taking into account its superior barrier properties.

Moreover, this review shows, for the first time, that there are effective R&D efforts to render PEF more sustainable, both in terms of green synthesis, as well as planning for its end-life and accounting for future problems regarding PEF disposal and accumulated waste debris in the environment. Both ROP and enzymatic assisted synthesis mediated by *Candida Antarctica* lipase provide positive signs of their use in PEF synthesis, although their broader application is still limited. With rational engineering, enzymes can be altered to display more selectivity and obtain higher polymerization yields compared to wild type ones. This is a powerful tool worth exploring in PEF synthesis to reach higher molecular weights. In terms of recycling, both mechanical recycling (with a higher degree of maturity), but also chemical recycling prompted by enzymes, were demonstrated to be adequate strategies. Special efforts toward making PEF with tuned degradability come from the modification of PEF by copolymerization with aliphatic moieties. Composting tests show fast PEF degradation, although biodegradation under relevant environmental conditions are lacking. In the next years, the use of other renewable-based monomers related to FDCA, such as thiophene derivatives, are expected to reach polymer research and technology developments and will modulate future polymers.

## Author Contributions

AFS, AJS, BA, DM, HH, IP, KL, NG, NS, and RZ summarized the information and prepared the manuscript. AFS organized the information in the manuscript and supervised the whole process. All authors contributed to the article and approved the submitted version.

## Conflict of Interest

The authors declare that the research was conducted in the absence of any commercial or financial relationships that could be construed as a potential conflict of interest.
